# Travel intentions of travelers in the COVID-19 context: The moderation of fear of COVID-19

**DOI:** 10.3389/fpsyg.2023.1136465

**Published:** 2023-03-02

**Authors:** Ruonan Tu, Sung Kyu Park, Yi Ding

**Affiliations:** Department of International Trade, Changwon National University, Changwon, Republic of Korea

**Keywords:** perceived value, perceived risk, social interaction, COVID-19, satisfaction

## Abstract

**Introduction:**

The spread of COVID-19 pandemic in early 2020 has significantly affected the tourism industry. Most current tourism research on emergencies focuses on issues such as the revitalization of the tourism economy. However, research on aspects such as visitor perception has not received sufficient attention, This study contributes to the literature by analyzing the effects of social interactions, multidimensional perceived value, fear of COVID-19, and age on travelers’ travel intentions during the COVID-19 pandemic.

**Method:**

This study constructs a structural equation model, formulates the corresponding hypotheses, investigates Chinese travelers, and verifies the moderating effect of COVID-19 fear.

**Results:**

All of the proposed hypotheses were verified. The three dimensions of perceived value and satisfaction had a significant mediating effect in the relationship between perceived quality and travel intention, and that fear of COVID-19 had a significant moderating effect in the relationship between satisfaction and travel intention. With the moderation of fear of COVID-19, age had a significantly negative effect on travel intention.

**Discussion:**

Given extant research demonstrating that both math activities and math talk predict children’s math skills, our results stress the need for multimethod studies that differentiate among these HME opportunitiesThe findings confirmed a significant mediating effect of the three dimensions of perceived value and satisfaction on perceived quality and travel intention. fear of COVID-19 had a significant moderating effect on satisfaction and travel intention. In addition, age had a significant negative effect on travel intention under the moderation of fear of COVID-19; thus, travel intention decreases with age.

## 1. Introduction

The spread of COVID-19 pandemic in early 2020 has significantly affected the lives of people worldwide. In particular, the tourism industry has suffered a huge impact, making it difficult to resume normal operations in the short term. In recent years, public health emergencies that affect tourism have begun to receive widespread attention from scholars. For example, in 2003, when SARS hit China and the world tourism industry, the number of outbound trips dropped dramatically from 702.6 million in 2002 to 694 million in 2003 (Zheng et al., 2021). Compared to SARS 2003, the impact of COVID-19 is more severe. With more than 80 countries and territories imposing travel restrictions in the early 2020s, including border closures, entry and exit bans, visa restrictions, and flight groundings (Think Global Health, 2020), the global tourism industry has taken a huge hit.

Most current tourism research on emergencies focuses on issues such as the revitalization of the tourism economy. However, research on aspects such as visitor perception has not received sufficient attention (Wu et al., 2021; Baloch et al., 2022; Spencer and Spencer, 2022). To some extent, how tourists perceive COVID-19 and what behaviors they adopt may have a long-term impact on the tourism recovery trends (Wen et al., 2020). Therefore, to facilitate an effective recovery of tourism, it is necessary to focus on customers’ perceived quality, perceived risk, perceived value, social interaction, satisfaction, and travel intentions of the destination. Considering that the impact of the pandemic is set to continue, it may take time for the tourism industry to return to its previous level during this period (Ntounis et al., 2021). Therefore, the purpose of this study is to investigate various factors affecting travelers’ intentions during the pandemic, and to find effective countermeasures to alleviate the impact of the pandemic.

The downturn in the general environment has posed challenges for destinations, which necessitates the need to address issues such as ways to attracting potential customers and maintaining existing customers. Perceived value is closely related to the social exchange theory of marketing and is the key to managing business-consumer relationships and consumers’ consumption decisions (Peng et al., 2019). It is considered one of the most important factors in attracting consumers and ensuring the competitive advantage of service providers (Hanaysha, 2018). Because the definition of perceived value varies depending on the situation, this study considers perceived value as a result of the consumption experience. According to Zeithaml (1988), perceived value is consumers’ overall evaluation of what is received compared to what is given.

Most previous studies classify perceived value into one (Sarstedt et al., 2020; Min, 2022; Qiao et al., 2022) or two dimensions (Alam et al., 2022; Guo and Li, 2022; Phuthong, 2022), and only a few studies classify perceived value into multiple dimensions. Therefore, this study analyzes perceived value in product and service evaluations in three dimensions based on previous studies (Sweeney and Soutar, 2001; Currás-Pérez et al., 2018; Bae et al., 2019; Meeprom and Silanoi, 2020). Specifically, it is divided into emotional, social, and economic value, and consumers’ consumption characteristics can be understood in detail, which is important for the success of product and service providers. Perceived value is a core component of a company’s strategy in attracting and sustaining consumers and is a necessary component concept for predicting consumer travel intentions. Therefore, perceived value is included as a core construct in this study’s model.

As mentioned in previous studies, perceived quality and perceived risk are also considered important antecedent variables of perceived value. Perceived quality is the customer’s perception of the overall quality or advantage of a product or service relative to its intended purpose (Aaker, 1991). Good perceived quality can enhance customer perceptions of the perceived value of a tourism destination.

Perceived risk refers to consumer perceptions of the uncertainty and adverse consequences of engaging in an activity (Wolff et al., 2019). Şen Küpeli and Özer (2020) consider perceived risk an important variable in customer decision-making because it reflects the perception of uncertainty and adverse consequences of the purchased product. An increased perceived risk can cause people to doubt their need to buy (Xu and Jackson, 2019). Therefore, reducing perceived risk helps increase consumer-perceived value (Wang et al., 2019).

Perceived good quality not only increases the customer’s perceived value of the destination (Ahn and Thomas, 2020), but also reduces perceived risk (Wang et al., 2019). Therefore, this study deepens the understanding of the relationship between consumer satisfaction and travel intention by identifying the relative influence of antecedent variables that affect the perceived value dimension, and by examining the direct and mediating effects of the perceived value dimension.

Recently, researchers have focused on determining the relationship between perceived value and antecedent (e.g., perceived quality, perceived risk, and social interactions) and outcome variables (e.g., satisfaction). However, few studies have been conducted to understand the relationship between social interactions and perceived value. Social interaction is an important factor in consumer behavior decisions, and numerous scholarly studies support this view (Yang, 2019; Hamilton et al., 2020; Moon and An, 2022). Luo et al. (2019) defined social exchange as the human interaction between customers. With the rapid growth of the Internet, tourists can not only experience face-to-face contact with different people at their destinations but also interact digitally with others through various social media platforms while traveling (Fan et al., 2019). Meeting the social needs of travelers can significantly enhance their perceived value (Zhang et al., 2017). Existing explorations of the relationship between social interactions and perceived value in the context of the pandemic are not sufficiently comprehensive. Most studies on social interactions are limited to social media and do not consider face-to-face encounters (Xu et al., 2021; Yılmazdoğan et al., 2021; Wang et al., 2022). Under special circumstances such as the COVID-19 pandemic, social activities are gradually decreasing due to various reasons such as infection and death risks. In this context, social interactions should be enhanced to form positive attitudes toward tourist destinations and increase consumers’ travel intentions. To this end, this study extends the existing research by incorporating social interactions, which is rarely mentioned in the COVID-19 context, into the research model.

And perceived value is generally considered to be an important factor in improving customer satisfaction (Chen and Lin, 2019). Existing research also often mentions that customer satisfaction is the most important factor for the success of product and service providers (Gajewska et al., 2019). According to research findings, higher perceived value leads to higher customer satisfaction, increased customer loyalty, and customer retention (Samadou and kim, 2018). Customer satisfaction is the customer’s overall evaluation of product performance (Johnson and Fornell, 1991).

Customer satisfaction is a post-purchase attitude formed by comparing customer expectations with perceived outcomes. When travelers are satisfied with an intended destination, their intention to visit is also enhanced. Travelers’ past experiences can influence their travel intentions, which, in turn, can significantly impact their future travel decisions (Jang and Namkung, 2009). However, most studies with Chinese respondents neglect the relationship between perceived value and customer satisfaction in COVID-19 context. Generally, when consumers plan a trip, they expect perceived value in advance. If these expectations are met, the possibility of continued consumption increases, which increases customer satisfaction and ultimately increases travel intentions.

This study hypothesizes that, in the context of the COVID-19 pandemic, the level of fear of COVID-19 has an impact on travel intentions. Fear of COVID-19 refers to negative emotions such as depression and anxiety due to the awareness of the possible outcomes of COVID-19, such as coronavirus infection (Ahorsu et al., 2020). With the normalization of COVID-19, people are inevitably exposed to it and travel. The COVID-19 pandemic has severely affected the tourism industry, especially the industry’s profitability. However, systematic research on fear of COVID-19 in the field of tourism remains insufficient. Therefore, it is necessary to comprehensively explore the role of fear of COVID-19. Most existing studies consider fear of COVID-19 as an independent variable, and its role as a moderating variable is neglected. Besides, even if the existing literature considers fear of COVID-19 as a moderating variable, most of them mainly focus on mental health (Fitzpatrick et al., 2020; Martínez-Lorca et al., 2020; Satici et al., 2020). Therefore, in the context of the COVID-19 pandemic, this study verifies the role of fear of COVID-19 as a moderating variable between consumer satisfaction and travel intentions.

In summary, this study verifies the structural relationships between perceived quality, perceived risk, social interaction, three-dimensional perceived value, consumer satisfaction, and travel intention and assesses the role of fear of COVID-19 as a moderating variable between satisfaction and travel intention. The objective of this study was to explore the structural relationships among perceived quality, perceived risk, social interaction, perceived value, satisfaction, and travel intention. More specifically, this study aimed to (1) investigate the effects of perceived quality, perceived risk, and social interaction on perceived value; (2) investigate the relationship between perceived value factors and satisfaction; (3) explore the relationship between satisfaction and travel intention; (4) test whether perceived value and satisfaction mediate the relationship between perceived quality and travel intention; (5) test the moderating effect of fear of COVID-19 on the relationship between satisfaction and travel intentions; and (6) examine the effect of age as a control variable on travel intention based on the moderating effect on fear of COVID-19.

This study extends the previous research in four ways. First, unlike previous studies, this study investigates Chinese travelers and compares the MZ era with the non-MZ generations. Second, the moderating effect of the fear of COVID-19 is examined. In addition, the impact of the need for socialization on travelers’ perceived value in this context is also discussed. Finally, this study clarifies the relationship between perceived risk, perceived value, satisfaction, and travel intention by examining the mediating role of perceived value and satisfaction.

The remainder of this paper is organized as follows. Section 2 briefly introduces the theoretical background, presents the hypotheses, and constructs the model from the previous study. Section 3 describes the questionnaire, data collection methods, and descriptive statistics. Section 4 tests the hypotheses and analyzes the results. Section 5 discusses the theoretical and practical implications of the findings and explains the limitations of the study and future research directions. Section 6 summarizes this research.

## 2. Theoretical background and hypothesis formulation

During the COVID-19 pandemic, tourism was the most affected industry, with the devastating impact of coronavirus on both destinations and tourism establishments (Pappas, 2021). Tourism is an essential pillar of the tertiary industries, and travel is a critical need of life and a solace for people. The recovery and revitalization of the tourism industry have become important issues that cannot be delayed.

### 2.1. Perceived quality

Providing high-quality products has become an important strategy for service firms to gain a competitive advantage (Konuk, 2019). This study adopts Zeithaml’s (1988) definition of perceived quality. Perceived quality is defined as consumers’ judgment of the overall excellence or superiority of a product (Zeithaml, 1988, p. 3). Before obtaining a service, customers create expectations based on their personal needs, past experiences, third-party recommendations, and service provider advertising. After purchasing and using the service, customers compare the expected quality with the quality they receive (Lopes et al., 2019).

For the service industry, especially the tourism industry, understanding customer perceptions of destination quality and improving customers’ perceived value are critical. Perceived value is a dynamic construct based on pre-purchase experience and perceived experience during and after use (Sánchez et al., 2006, p. 1). Consumer-perceived value is essential in the tourism industry, and enhancing customer-perceived value is an important means to maintaining a sustainable competitive advantage (Hanaysha, 2018; Chen et al., 2019; Itani et al., 2019). Additionally, perceived value is a critical factor for consumers to make choices (Konuk, 2019).

Babin et al. (1994) divided perceived value into two dimensions: hedonic and utilitarian. Sweeney and Soutar (2001) divided perceived value into four dimensions (emotional, social, quality, and economic). Bradley and Sparks (2011) argued that perceived value is determined by multiple factors, and that consumers predict and obtain value through many sources. This study draws on Currás-Pérez, Jeong-Tae Bae, and Meeprom’s three-dimensional classification and uses three dimensions: emotional, social, and economic value. Emotional value is the utility gained from a feeling or emotional state generated by a product. Social value (enhanced social self-concept) is derived from the utility of a product’s ability to improve its social self-concept. Economic value is the utility gained from the product because of the reduced short- and long-term costs.

Monroe (1990, p. 46) defined perceived value as “the trade-off between the perceived quality or benefit that consumers have in a product and the perceived sacrifice of the price they pay;” The perceived quality of special events can directly affect social, economic, and emotional value (Meeprom and Silanoi, 2020). Suttikun and Meeprom (2021) show a positive relationship between customers’ perceived quality and perceived value when customers purchase souvenirs. Widodo and Maylina (2022) conclude that the perceived value of airline passengers in all dimensions is directly influenced by perceived quality. Therefore, this study assumes that the perception of perceived value dimension affects perceived quality and proposes the following hypothesis:

*H1.* Perceived quality positively affects perceived value.

*Hypothesis 1-1* Perceived quality positively affects perceived emotional value.

*Hypothesis 1-2* Perceived quality positively affects perceived economic value.

*Hypothesis 1-3* Perceived quality has a positive effect on perceived social value.

### 2.2. Perceived risk

Perceived risk theory is first introduced to consumer scenarios by Bauer (1960). According to Bauer (1960), consumers face corresponding risks in making behavioral decisions because purchases produce outcomes that consumers cannot fully determine and anticipate; thus, they are likely to have unpleasant consequences. Scholars have different understandings of the definition of perceived risk. Some scholars believe that perceived risk is a feeling of uncertainty in the product purchase process, because consumers cannot predict the outcome of their purchases and consequences (Derbaix, 1983). Some scholars believe that perceived risk should be composed of performance, financial, time, social, and security and privacy risks (Lee, 2009). This study adopts Bauer’s (1960) definition of perceived risk, which states that “the perception of uncertainty arising from the traveler’s inability to determine the outcome of his or her decision and the resulting adverse consequences in the product selection process.”

With the advancement of transportation and contact methods, tourism has been booming in recent years, and many travel and trading platforms have emerged. These developments, which have allowed travelers to purchase tickets and specialties offline and directly online, have increased the complexity of perceived risks arising from the shopping process. As you can see, the new environment and channels have also created new perceived risks for travelers. In recent studies, the concept of perceived risk is often considered an explanatory variable that influences customer behavior (Holbrook, 1994). To understand this behavior, perceived value and risk also frequently appear together in relevant studies (Chen et al., 2017; Şen Küpeli and Özer, 2020).

Hansen et al. (2018) consider perceived risk as a decisive antecedent in end-user decision-making. Tzavlopoulos et al. (2019) argue that a high level of perceived quality leads to higher satisfaction and perceived value, which reduce perceived risk and ultimately influence consumers’ adoption of positive consumption behaviors. Tzavlopoulos et al. (2019) also verify that an increase in perceived risk leads to a decrease in perceived value from an e-commerce perspective. Uslu and Karabulut (2018) show that in Fethiye (TR), destination perceived risk has a negative impact on travelers’ perceived value. In addition, consumers’ perceived risk in their choice of consumption channel may also inhibit their perceived value of the product or service (Broekhuizen and Jager, 2004). In most studies, on the issue of perceived risk on perceived value, it has been agreed that perceived risk has a negative impact on perceived value (Tzavlopoulos et al., 2019; Li et al., 2020; Xie et al., 2021). Therefore, this study assumes that the perception of perceived value dimension affects perceived risk and proposes the following hypothesis:

*H2.* Perceived risk has a negative impact on perceived value.

*Hypothesis 2-1* Perceived risk negatively affects perceived emotional value.

*Hypothesis 2-2* Perceived risk has a negative impact on perceived economic value.

*Hypothesis 2-3* Perceived risk has a negative impact on perceived social value.

### 2.3. Social interaction

Existing literature suggests that interactions between customers affect their evaluation of the service experience (Wu, 2008) and their product choice (Murphy, 2001). Georgi and Mink (2013, p. 11) considered social interaction as “any type of personal or group social interaction that customers encounter when acquiring and consuming goods and services.” Sunitiyoso et al. (2011) found that social interaction is the most direct interaction that occurs through informative communication between travelers about their travel experiences (choices and their outcomes) or intentions (communication here encompasses all possible social media, as well as face-to-face, phone, video, and SMS communication). The enjoyment gained from such interactions increases consumers’ loyalty and sense of belonging to their travel destination (Koh et al., 2003). It also enhances the interactivity between users and increases perceived value, which enhances the intention to continue using the product (Zhang et al., 2017). This study follows Georgi and Mink’s conceptual definition of social interactions.

Zhang et al. (2017) show that social interactions in social network services (SNS) have a positive impact on perceived value. Social interactions between viewers and anchors also affect viewers’ (consumers’) perceived value (Chen and Lin, 2018). Hussein et al. (2018) conclude that the hospitality industry can effectively increase perceived value and thus retain customers by improving their social interactions. The above studies have also suggested that social interactions play an important role in the dynamics of consumer behavior. Therefore, this study assumes that the perception of perceived value dimension affects social interaction quality and proposes the following hypothesis:

*H3.* Social interactions positively affects perceived value.

*Hypothesis 3-1* Social interactions positively affects perceived emotional value.

*Hypothesis 3-2* Social interaction positively affects perceived economic value.

*Hypothesis 3-3* Social interaction has a positive effect on perceived social value.

### 2.4. Perceived value

Owing to the diversity of customer needs, fierce competition, and rapid technological development, many service companies try to deliver the best customer value to gain a competitive edge (Woodruff, 1997). Many researchers agree that creating the best customer value is a critical goal for any company. Delivering the best customer value can be regarded as an important factor for the success of hospitality companies (travel industry; Yrjölä et al., 2019; An and Han, 2020; Fitriasari, 2020). Zeithaml (1988. p. 3) defined customer perceived value as the overall evaluation of the benefits customers perceive from the product or service weighed against the costs, removing the fact that the greater the difference between benefits and costs, the greater the customer perceived value.

Perceived value, the most important measure of a company’s competitive advantage, is an important predictor and key determinant of customer satisfaction and loyalty (Lee et al., 2007). Customer satisfaction is usually studied as a unidimensional construct that measures overall satisfaction with the service organization, which is the result of a combined judgment of all interactions with the organization (El-Adly, 2018).

Matsuoka et al. (2017) find that an increase in travelers’ perceived value of a destination increases satisfaction. Lu and Wang (2020) find that, from the travel perspective, an increase in travelers’ perceived value increases customer satisfaction to some extent. Using Daegu city tourist destinations as an example, Peng and Jiang (2022) find that perceived value has a significant positive effect on customer satisfaction. Moreover, in the COVID-19 context, most existing tourism studies on perceived value are conducted in a single dimension, and research on multidimensionality is limited; we explore multidimensional perceived value. Therefore, this study assumes that the perception of perceived value dimension affects customer satisfaction and proposes the following hypothesis:

*H4.* Perceived value has a positive impact on satisfaction.

*Hypothesis 4-1* Perceived emotional value positively affects satisfaction.

*Hypothesis 4-2* Perceived economic value positively affects satisfaction.

*Hypothesis 4-3* Perceived social value has a positive impact on satisfaction.

### 2.5. Customer satisfaction

Satisfying customers is the goal of marketing (Thakur, 2019) because it can bring long-term benefits, such as positive reputation and customer loyalty, to the product (Torabi and Bélanger, 2021; Lin et al., 2022). Customer satisfaction is the overall evaluation of a product obtained by the customer through its performance to date (Johnson and Fornell, 1991). Satisfaction stems from the comparison between consumers’ expectations of a product’s performance and its actual performance (Oliver, 1980). This is a follow-up study based on Oliver’s (1980)definition of satisfaction.

Tourist satisfaction affects travel intention. As an important tool for evaluating experiences, it profoundly influences destination choice, consumption of products and services, future travel intentions, and recommendations to others (Shafiee et al., 2016). Boulding et al. (1993) defined use intention as an individual’s will and belief in expressing a specific future behavior after consumers form value for an object.

Bayih and Singh (2020) explore the issue of intra-country travel and find satisfaction to be an important antecedent of travel intention. He and Luo (2020) show that the higher the satisfaction of travelers during snow and ice travel, the higher their intention to travel. Satisfaction with VR travel positively effects travel intention (An et al., 2021). The above studies provide evidence that satisfaction is a strong indicator of action intention, repeat purchase, and recommendation of a product or service. Therefore, traveler satisfaction plays a prominent role in travel intention, so this paper proposes the following hypothesis:

*H5.* Satisfaction has a positive effect on travel intention.

### 2.6. The mediating role of perceived value and satisfaction

The verification of the mediating effect of perceived value and customer satisfaction is important in this study. In this study, the direct effect between perceived quality and travel intention is not verified; although, previous studies find a direct effect (Hou et al., 2021; Wasaya et al., 2021; Hashish et al., 2022). Research on the mediating effect of perceived value and customer satisfaction on the relationship between perceived quality and travel intention during the COVID-19 pandemic is insufficient. Therefore, this study verifies the serial mediating effect of perceived value and customer satisfaction on the relationship between perceived quality and travel intention.

Perceived quality drives the perceived value of customers; if customers invest less time, money, and effort than they receive in perceived quality, customers receive a higher perceived value (Hapsari et al., 2016). In other words, the higher the quality of perception, the higher is the perceived value of the customer. Keshavarz and Jamshidi (2018) validate the mediating role of perceived value in perceived quality and customer behavior from the perspective of the hospitality industry. Meanwhile, Cempena et al. (2021) validate the role of perceived value as a mediating variable between perceived quality and tourist behavior from the perspective of the Gianyar Regency tourist attraction in Bali, Indonesia. Gómez-Carmona et al. (2022) also verify the mediating role of perceived value between service quality and tourist behavior from the perspective of health services. The above studies have examined the mediating role of perceived value, perceived quality, and consumer behavior. This study further explores the mediating role of perceived value between perceived quality and travelers’ satisfaction, based on prior research.

Jeong and Kim (2019) concluded that good perceived value contributes to customer loyalty and retention. Khawaja et al. (2021) verify the mediating role of customer satisfaction in private universities in Lebanon between customer-perceived quality and behavioral intentions. Rahardja et al. (2021) confirm the mediating role of satisfaction between perceived value and customers’ intention to act in the context of business activities in social media. The finding that satisfaction plays a mediating role in perceived value and consumer behavior is also supported by the above-mentioned studies. However, in the COVID-19 context, research on the serial multiple mediation model of perceived value and customer satisfaction in the relationship between perceived quality and travel intention is insufficient. Therefore, this study examines the multiple mediating roles of perceived value and customer satisfaction in the relationship between perceived quality and travel intention and proposes the following hypothesis:

*H6.* Perceived value and travelers’ satisfaction play a serial mediating role in the relationship between perceived quality and tourism intention.

*Hypothesis 6-1* Perceived emotional value and travelers’ satisfaction play a serial mediating role in the relationship between perceived quality and tourism intention.

*Hypothesis 6-2* Perceived economic value and travelers’ satisfaction play a serial mediating role in the relationship between perceived quality and tourism intention.

*Hypothesis 6-3* Perceived social value and travelers’ satisfaction play a serial mediating role in the relationship between perceived quality and tourism intention.

### 2.7. The moderating role of fear of COVID-19

Fear of COVID-19 is a negative emotional state of depression and anxiety due to the awareness of possible outcomes associated with COVID-19, such as infection with coronavirus (Ahorsu et al., 2020). Fear of COVID-19 increases anxiety about going out, and thus significantly decreases consumer behavioral intentions (Apaolaza et al., 2022). Even if they are satisfied with their destination, travelers will reduce their travel intentions under the influence of fear of COVID-19 (Rehman et al., 2021). Several tourism marketing scholars have explored the moderating role of fear of COVID-19 in the relationship between attitudes (satisfaction, engagement) and behavior (Liu H. et al., 2021; Rather, 2021; Akhrani et al., 2022). However, most studies using fear of COVID-19 as a moderating variable have also focused on the mental health domain (Gasparro et al., 2020; Dymecka et al., 2021; Soto-Sanz et al., 2021). This study builds on the fear of COVID-19 definition by Ahorsu et al. (2020).

Previous studies have shown that age affects intention (Na et al., 2021; Watanabe et al., 2021; Herzallah et al., 2022); and in this study, age was included as a control variable (Hien et al., 2021; Klaver et al., 2021; Saari et al., 2021). Research on the moderating role of COVID-19 fear in the relationship between satisfaction and travel intention is lacking; therefore, this study proposes the seventh hypothesis:

*H7.* Fear of COVID-19 plays a moderating role in the relationship between satisfaction and travel intention.

**Figure 1:**
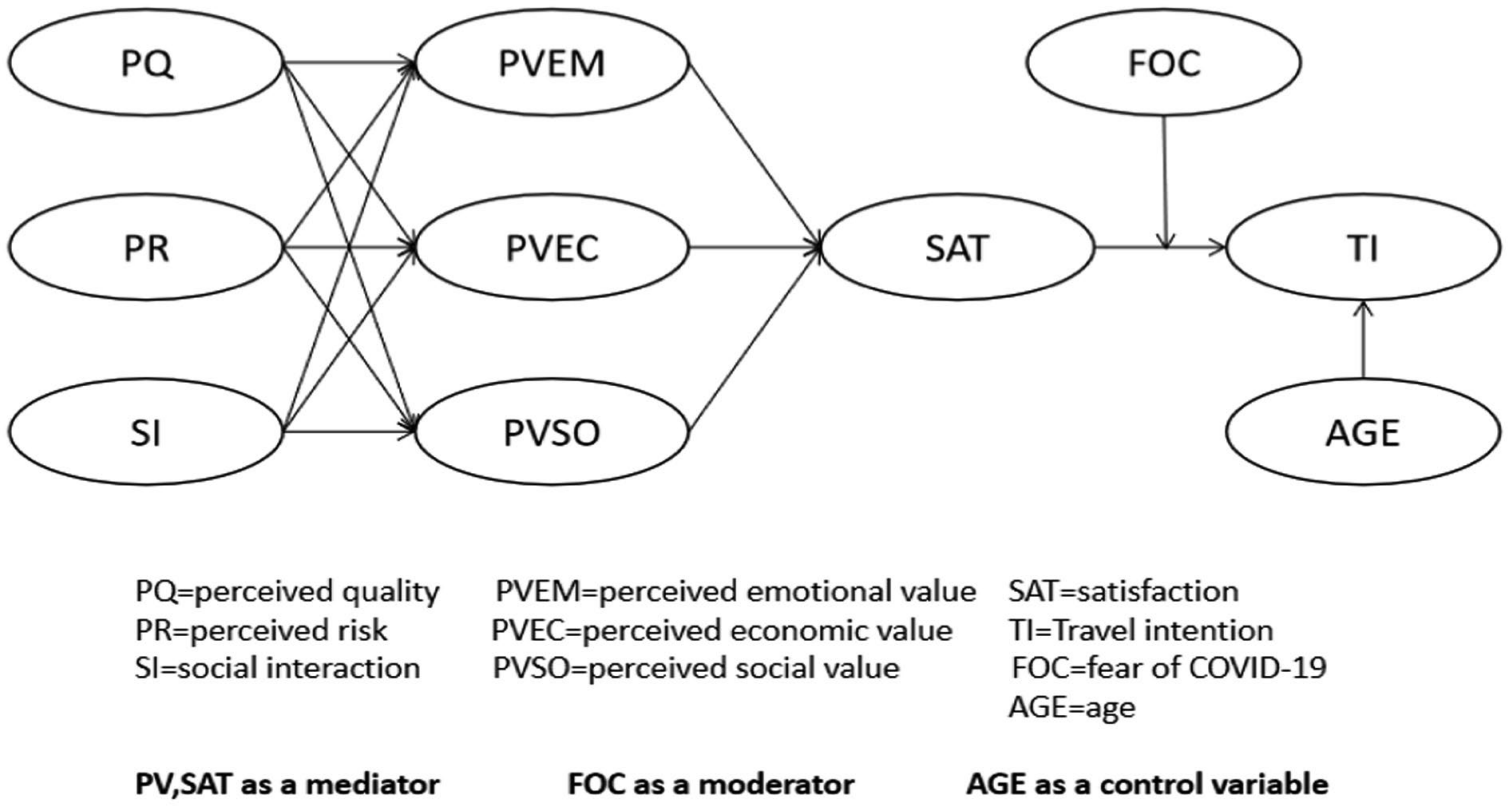
Research model.

This study constructs the research model described above (see Figure 1).

## 3. Methodology

### 3.1. Questionnaire design

The items measured for each construct in this study were adapted from previous research and included perceived value (emotional, social, and economic), perceived quality, perceived risk, social interaction, satisfaction, and travel intention. A 5-point Likert scale was used in this study, with 1 indicating strong disagreement and 5 indicating strong agreement. These scales were initially written in English, and after consulting experts and making corrections, the questionnaire was finally translated into Chinese and distributed to the respondents.

Perceived quality was measured using the scale of Chi et al. (2019). For perceived risk, the scales of Wang et al. (2019) and Şen Küpeli and Özer (2020) were used. For perceived value, the scales of Peng et al. (2019) and Meeprom and Silanoi (2020) were used. For socialization, the scales of Lee et al. (2019) and Jasrotia et al. (2022) were used, and the scales of Konuk (2019), Yan et al. (2021), and Liu P. et al. (2021) were used for satisfaction. Chi et al.’s (2019) scale was used for travel intention. The scale developed by Ahorsu et al. (2020) was used to assess the fear of COVID-19.

### 3.2. Data collection and descriptive statistics of the sample

This study used an online cross-sectional survey. Convenience sampling was conducted based on the topic of this study. Given that the purpose was to analyze the travel intentions of Chinese travelers in the context of the COVID-19 pandemic, participants had to meet the following requirements: (1) be at least 18 years of age and (2) need to have traveled in the last 3 years.

The proposed hypothesis is tested using a questionnaire. Fifty Chinese respondents with a history of traveling in 3 years after the outbreak of the COVID-19 pandemic were first screened as pre-test respondents. Inappropriate questions were then removed, and a final questionnaire was created. The survey was conducted from 1 August to 20 August 2022, and questionnaires were distributed and collected through a questionnaire company (wenjuanxing). A total of 518 questionnaires were returned; 15 invalid questionnaires were removed and 503 were finally used in this study. The demographic characteristics of the participants were as follows.

The results showed that the proportion of female respondents (51.49%) was higher than that of male respondents (48.51%). In addition, the age distribution of respondents was relatively balanced, with respondents aged 18–40 (53.08%) years and over 41 (46.92%) years. Approximately 7.8% of the respondents were in the 18–20 age group, 23.5% were in the 21–30 age group, 21.5% were in the 31–40 age group, 21.9% were in the 41–50 age group, 24.7% were in the 51–60 age group, and 0.6% were aged 61 and above. Most respondents had a college degree or higher (61.83%). The income distribution of the respondents is as follows: monthly income of 3,000 RMB and below (16.90%), 3,001–4,000 (7.55%), 4,001–5,000 (14.51%), 5,001–6,000 (16.90%), 6,001–7,000 (11.73%), and 7,001 and above (32.41%). The number of married respondents (72.17%) was higher than that of unmarried respondents (27.04%), while others accounted for 0.79% of the respondents. In addition, the occupational distribution of the respondents was as follows: students (19.09%), enterprise staff (26.44%), self-employed (10.34%), civil servants (18.69%), housewives (2.58%), freelancers (15.31%), retirees (3.18%), and others (4.37%). Of these, 354 (70.38%) had traveled within the last year, 121 (24.06%) within 2 years, and 28 (5.56%) between 2 and 3 years.

## 4. Results and data analysis

### 4.1. Normality test

The data were analyzed by structural equation modeling using AMOS 25. This study set up a measurement and structural model using ML estimation methods to verify the normality of the underlying assumptions of ML estimation. The results showed that the skewness of the variables ranged from 0.688 to −1.319, and the kurtosis ranged from −1.165 to 2.035, indicating that the basic assumptions of skewness ±3 and kurtosis ±7 were satisfied, and the sample was normal (Kline, 2015).

### 4.2. Common method bias testing

Harman’s (1967) one-factor test was used to test for common method bias in the data. The total variance extracted by one factor was 36.812%, which is lower than 50%. Therefore, there was no obvious common-method bias.

In addition, all dimension indicators (nine dimensions, 27 questions) were included in the one-factor confirmatory factor analysis. When there is common method bias, the model value of the one-factor confirmatory analysis is higher than that of the multi-factor model. The one-factor validation model’s values (χ^2^/df = 16.668, GFI = 0.542, AGFI = 0.466, CFI = 0.486, SRMR = 0.1207, and RMSEA = 0.177) were much lower than those of the multi-factor model (χ^2^/df = 1.882, GFI = 0.926, AGFI = 0.903, CFI = 0.974, SRMR = 0.0294, and RMSEA = 0.042), further indicating that there was no obvious common method bias in this study (Table 1).

**Table 1:** Model comparison.

Model	χ^2^	DF	△χ^2^	△DF	*p*
One-factor	5400.428	324	4858.415	36	0.000
Multi-factor	542.013	288			

The difference between the one-factor model and multi-factor model’s Chi-square test was compared and reached a significant level (*p* < 0.05). The Chi-square of the two models was significantly different, indicating that there was no severe common method bias in this study (Mossholder et al., 1998).

### 4.3. Validity and reliability of measurement

Cronbach’s alpha was used to assess the internal consistency of the scale, and a value of above 0.7 was considered sufficient (Bagozzi and Yi, 1988). Cronbach’s α of all items exceeded the criterion of 0.8 from 0.831 to 0.945, indicating that this study had sufficient reliability.

Hair et al. (2010) proposed that models should be evaluated on the basis of convergent and discriminant validity. Convergent validity should be evaluated using the CR and AVE values for each construct. As shown in Table 2, the CR of all the constructs was between 0.835 and 0.945, all of which met the criterion of 0.7, and the AVE of all the constructs also met the criterion of 0.5 between 0.630 and 0.852 (Fornell and Larcker, 1981). In addition, Hair et al. (2011) suggested that the factor loadings for each item should be greater than 0.60 to establish convergent validity, while the factor loadings for all the constructs in this study were between 0.724 and 0.963, which also met the criteria. Therefore, the measurement model used in this study had an acceptable level of convergent validity.

**Table 2:** Results of confirmatory factor analysis.

	Items	UNSTD	S.E.	*t*-value	STD	CR	AVE
PQ	PQ1	0.971	0.050	19.597***	0.797	0.870	0.690
	PQ2	1.000			0.882		
	PQ4	0.970	0.049	19.887***	0.811		
PR	PR1	0.801	0.051	15.664***	0.724	0.835	0.630
	PR3	1.000			0.895		
	PR5	0.833	0.052	16.061***	0.751		
SI	SI2	1.000			0.912	0.888	0.726
	SI3	0.662	0.031	21.588***	0.803		
	SI4	0.962	0.042	22.661***	0.837		
PVEM	PVEM2	0.890	0.043	20.727***	0.785	0.884	0.718
	PVEM3	1.000			0.919		
	PVEM4	0.890	0.040	22.17***	0.832		
PVEC	PVEC2	0.869	0.041	20.963***	0.765	0.894	0.739
	PVEC3	0.987	0.039	25.421***	0.896		
	PVEC4	1.000			0.910		
PVSO	PVSO1	0.919	0.049	18.753***	0.820	0.860	0.671
	PVSO3	0.879	0.048	18.433***	0.797		
	PVSO4	1.000			0.840		
SAT	SAT1	0.959	0.037	26.054***	0.866	0.911	0.774
	SAT3	1.000			0.903		
	SAT4	0.998	0.038	26.214***	0.870		
TI	TI1	0.879	0.025	34.999***	0.889	0.945	0.852
	TI2	1.000			0.963		
	TI3	0.930	0.024	38.09***	0.915		
FOC	FOC1	0.971	0.040	24.357***	0.858	0.900	0.749
	FOC2	1.000			0.899		
	FOC3	1.090	0.046	23.697***	0.839		

*** *p* < 0.001.

In addition, multicollinearity test was also performed. Tolerances ranged from 0.774 to 1, all greater than 0.2. VIF ranged from 1 to 1.292, all of which were less than 5, thus ruling out the possibility of multicollinearity (Hair et al., 2010).

The Heterotrait Monotrait Ratio (HTMT) has been shown to yield greater specificity and sensitivity (97–99%) than Fornell and Larcker’s (1981) criterion (20.82%; Ab Hamid et al., 2017). Therefore, HTMT criteria were used in the study. The HTMT method specifically assesses discriminant validity by comparing the HTMT values between two constructs, in which the HTMT value between the two factors should be less than 0.85. As shown in Table 3, the results of HTMT ratio ranged from 0.106 to 0.711, indicating that all constructs were independent of each other. Therefore, all constructs in this study had sufficient discriminant validity.

**Table 3:** Discriminant validity (HTMT criteria).

	PQ	PR	SI	PVEC	SAT	TI	PVSO	PVEM	FOC
PQ									
PR	0.346								
SI	0.298	0.166							
PVEC	0.498	0.284	0.297						
SAT	0.68	0.395	0.358	0.484					
TI	0.602	0.349	0.29	0.349	0.711				
PVSO	0.706	0.407	0.313	0.392	0.623	0.552			
PVEM	0.574	0.31	0.282	0.374	0.592	0.407	0.489		
FOC	0.348	0.4	0.106	0.227	0.389	0.302	0.398	0.297	

### 4.4. Hypothesis testing

The model fit of this study was as follows: χ^2^ = 518.621, χ^2^/df = 2.026(<3), RMR = 0.038(<0.05), RMSEA = 0.047(<0.05), which met the criteria. In addition, GFI = 0.924, AGFI = 0.904, NFI = 0.943, RFI = 0.933, IFI = 0.970, TLI = 0.965, and CFI = 0.970 were all above the standard of 0.9. Therefore, this theoretical model setting was acceptable.

The results of the path analysis revealed that all the hypotheses presupposed in this study were valid, and that there was a positive relationship between PQ and PVEM (H1-1: *β* = 0.537, *t* = 10.924, *p* < 0.001), PVEC (H1-2: *β* = 0.447, *t* = 8.935, *p* < 0.001), and PVSO (H1-3: *β* = 0.618, *t* = 12.583, *p* < 0.001). There were also negative relationships between PR and PVEM (H2-1: *β* = −0.096, *t* = −2.098, *p* < 0.05), PVEC (H2-2: *β* = −0.101, *t* = −2.134, *p* < 0.05), and PVSO (H2-3: *β* = −0.185, *t* = −4.271, *p* < 0.001). Positive relationships existed between SI and PVEM (H3-1: *β* = 0.122, *t* = 2.790, *p* < 0.01), PVEC (H3-2: *β* = 0.155, *t* = 3.412, *p* < 0.001), and PVSO (H3-3: *β* = 0.111, *t* = 2.715, *p* < 0.01). A positive relationship was found between perceived value (PVEM, PVEC, and PVSO) and SAT (H4-1: *β* = 0.324, *t* = 7.710, *p* < 0.001; H4-2: *β* = 0.203, *t* = 5.110, *p* < 0.001; H4-3: *β* = 0.420, *t* = 9.174, *p* < 0.001). There was also a positive relationship between the SAT and TI (H5: *β* = 0.704, *t* = 18.952, *p* < 0.001).

A phantom model (see Table 4) was used in this study to analyze the results of the indirect effects and statistical significance. PVEM and SAT (*p* < 0.000), PVEC and SAT (*p* < 0.001), and PVSO and SAT were all mediated by PQ and TI (*p* < 0.000). There was no 0 in the 95% CI, again validating the existence of a mediating effect.

**Table 4:** Standardized structural estimates (total sample).

Path		Standardized estimates	Standardized error	*t*-value
H1-1	PQ → PVEM	0.537	0.059	10.924***
H1-2	PQ → PVEC	0.447	0.064	8.935***
H1-3	PQ → PVSO	0.618	0.057	12.583***
H2-1	PR → PVEM	−0.096	0.046	−2.098*
H2-2	PR → PVEC	−0.101	0.050	−2.134*
H2-3	PR → PVSO	−0.185	0.042	−4.271***
H3-1	SI → PVEM	0.122	0.043	2.790**
H3-2	SI → PVEC	0.155	0.047	3.412***
H3-3	SI → PVSO	0.111	0.039	2.715**
H4-1	PVEM → SAT	0.324	0.036	7.710***
H4-2	PVEC → SAT	0.203	0.032	5.110***
H4-3	PVSO → SAT	0.420	0.041	9.174***
H5	SAT → TI	0.704	0.046	18.952***
Control variable	AGE → TI	−0.093	0.023	−2.760**
Mediating path		Indirect effect	95% Bootstrapping confidence intervals	*p*-value
H6-1	PQ → PVEM → SAT → TI	0.156	0.105 ~ 0.212	0.001
H6-2	PQ → PVEC → SAT → TI	0.081	0.046 ~ 0.122	0.001
H6-3	PQ → PVSO → SAT → TI	0.233	0.171 ~ 0.311	0.001
Moderating path		△df	△χ^2^	*p*-value
H7	SAT → TI (moderating effect of FOC)	1	4.601	0.032

* *p* < 0.1;

** *p* < 0.05;

*** *p* < 0.001.

Thus, PV and SAT not only play a direct role in influencing PQ to TI but also indirectly influence TI through their mediating effects on PV and SAT. Therefore, by improving the perception of destination quality, travelers’ perceived value and satisfaction can be further improved, thereby increasing their travel intentions.

This study argues that the hypothesis is supported if the change in the Chi-square (χ2) difference between the constrained and free models is above the cardinality standard threshold (Stone and Hollenbeck, 1989). When analyzing the effect of satisfaction on travel intentions, the analysis result showed that the constrained model was χ^2^ = 917.240 (df = 512), while the freedom model was χ^2^ = 921.841 (df = 513). The change in the difference in the constrained model for one degree of freedom, Δχ^2^ (1), was 4.601 (*p* = 0.032). The results were considered statistically significant, thus supporting H7 (see Table 4).

Tests for control variables were also conducted. Age was nominal scale, so this study classified 1–3 (18–40 years old) as 1 (MZ) and 4–6 (41+ years old) as 2 (non-MZ). Age had a statistically significant negative effect on TI (*β* = −0.093, *t* = −2.760, *p* < 0.01; see Table 4). Thus, the larger the Age, the lower the travel intention was. Age was also divided into two groups: MZ and non-MZ generation, with the MZ generation used as a reference group for the dummy variable under the FOC moderating effect. The results show that in the high FOC group, the non-MZ generation has a lower travel intention than the MZ generation. Therefore, we can assume that the older the age, the higher the fear of FOC, eventually leading to a lower travel intention.

## 5. Discussion

### 5.1. Findings

This study analyzes travelers’ travel intentions during the COVID-19 pandemic. It examines the structural relationships between perceived quality, perceived risk, social interaction, perceived value, satisfaction, and travel intention. First, the results reveal a significant positive relationship between all three dimensions of PQ and PV, with PQ having the most significant effect on PVSO. Our results are consistent with that of Choi and Kim (2013), Meeprom and Silanoi (2020), and Li and Shang (2020). Second, a significant negative relationship exists between the three dimensions of PR and PV, with PR having the most significant negative effect on PVSO. These findings are consistent with that of Şen Küpeli and Özer (2020), Li et al. (2020), and Xie et al. (2021). Third, a significant positive relationship exists between the three dimensions of SI and PV, with SI having the most significant effect on PVEC. Our results are consistent with that of Liu Y. et al. (2021), Li et al. (2021), and Zhang et al. (2022). Fourth, a significant positive relationship exists between all three dimensions of PV and SAT, with PVSO having the most significant effect on SAT. These findings are consistent with that of Choi and Kim (2013) and Rasoolimanesh et al. (2020). However, our results are slightly different from that of Prebensen and Xie (2017), whose results show that perceived economic value is insignificant. Finally, SAT has a positive impact on TI, which is consistent with the findings of He and Luo (2020), Pai et al. (2020), and Abbasi et al. (2021).

In addition, the results show that the three dimensions of PV and SAT have a significant mediating effect on the relationship between PQ and TI, and that FOC has a significant moderating effect on the relationship between SAT and TI. With the moderation of FOC, AGE has a significantly negative effect on TI, which is in line with the findings of Ayeh et al. (2013), Gemar et al. (2019), and Gu et al. (2021). Thus, the older the age, the lower the travel intention.

### 5.2. Theoretical implications

This study has four theoretical contributions to the literature.

First, previous studies have not specifically examined social interactions in the tourism field. Particularly, studies focusing on social interactions in the tourism field are limited despite the fact that the field values interaction with tourists. Because the Chinese government has had a zero-COVID policy for a longer period, travel has been extremely restricted during the zero-COVID period. Unlike the situation before zero-COVID, face-to-face interactions are lacking in the context of a newly crowned pneumonia-ridden China. This study not only expands the scope of the research by targeting tourists (or the tourism field), but also contributes theoretically to the expansion of existing research models.

Second, while studies on the effects of perceived value are widely available, most of them focus on one or two dimensions (e.g., utilitarian value and hedonic value). Therefore, based on Sheth et al. (1991), this study divides perceived value into multiple dimensions. It not only analyzes the direct effect of perceived value, but also explores its possible mediating effect, which should have a theoretical significance. In particular, further refining and differentiating perceived value, which is considered an important variable influencing tourists’ choice behavior, not only contributes to a clearer and more hierarchical understanding of actual travelers’ product and service purchasing behaviors and consumption processes, but also helps expand existing research in the field of tourism destinations.

Third, this study makes important methodological contributions to the literature. That is, the measurement items of fear of COVID-19 are validated with Chinese consumers. This study also uses the items developed by Ahorsu et al. (2020), which have been modified and revalidated for the Chinese pandemic situation; thus, contributing to the reliability and validity of the scale. Existing research on fear of COVID-19 has been conducted mainly in the mental health context. The present study not only extends the scope to the field of tourism, but also expands the existing research by adding an analysis on the moderating effect of fear of COVID-19.

Fourth, this study uses age as a control variable affecting travel intention in the context of the COVID-19 pandemic. Respondents are divided by age into the MZ generation (under 40 years) and non-MZ generation (after 40 years) based on the rationale that there are significant differences in product or service selection between the MZ and non-MZ generations. Future studies could expand age’s role as a moderating variable by dividing the subjects by age (MZ vs. non-MZ).

### 5.3. Practical implications

This paper contributes to the enhancement of tourism destinations from five perspectives.

First, the object of this study differs from previous studies in that it analyzes the differences in travel intentions of the MZ and non-MZ travelers during COVID-19, using FOC as a moderating variable. The results show that travel intention decreases as age increases under the moderation of FOC. The findings of this study can be used as a guide for tourist destinations to understand the behavior of travelers of different ages and improve traveler satisfaction and travel intention.

Second, the results show that perceived quality had a positive effect on all three dimensions of perceived value, with a significant effect on perceived social value. Social values reflect how well visitors are recognized, respected, and accepted by others (Caber et al., 2020). The experience of travel matches their social value expectations, emotional value, and economic value, similar to the results of prior studies (Fu et al., 2018; Kim and Thapa, 2018). Tourists consume tourism products to enhance their social image, as purchasing and participating in tourism can convey symbolic meaning to others. In addition, travelers often make travel choices through their own emotional appeals. It is possible that the perceived economic value of the travelers was not as high as that of the first two due to long-term pandemic isolation. To enhance the travel intentions of travelers, travel destinations should prioritize improving the perceived social value of travelers by conducting more interactive entertainment interactions so that travelers can fully interact with their peers, residents, and so on, and gain a sense of accomplishment in communication and collaboration. There is also a need to enhance the perceived emotional value of travelers, which can fully exploit the local characteristics of tourism resources to create a competitive advantage of differentiation and avoid uniformity. Destinations need to focus on enhancing the perceived economic value of travelers, and all aspects of food, lodging, transportation, shopping, and entertainment should be reasonably charged to show a simple style of honest management.

Third, the findings indicate that perceived risk has a negative effect on all three dimensions of perceived value, with perceived risk having the strongest negative effect on perceived social value, followed by perceived economic value and perceived emotional value. Because the current study did not compare PR with multidimensional PV (Tzavlopoulos et al., 2019; Xie et al., 2021), the results of this study could not be compared with those of existing studies. However, the overall results were the same and both showed a negative effect. Travelers’ perceptions of environmental risks reduce their perceived value, and the negative impact is the strongest because social values need to be realized in an overall free and safe environment. Therefore, to achieve the sustainable and healthy development of travel destinations, it is necessary to prevent risks. During the pandemic, it is important to pay attention to safe contact between travelers and service providers and to disinfect and sterilize public facilities in a timely manner to reduce the risk of COVID-19 infection. Simultaneously, the leakage of users’ personal information and additional unspecified charges during the transaction process should be minimized. A good image of tourist destinations should be established, transaction risks reduced, and the overall transaction experience improved to enhance the security management in tourist destinations, thereby motivating travelers’ intention to travel.

Forth, the results show that social interaction has a positive effect on all three dimensions of perceived value, with social interaction having the strongest effect on perceived economic value, followed by perceived emotional value and perceived social value. To enhance the experience of social interaction, travel destinations should not only focus on social business platforms but also strengthen the development of basic applications, especially in the new technological environment using artificial intelligence, big data, cloud computing, and virtual reality technologies to seek breakthroughs for the destination. Thus, destinations can satisfy the demand for interactive experiences in various forms, save time, and promote the sustainable development of tourist places. Additionally, different marketing strategies should be used for different customer groups. Travelers have different motivations and focus on different types of interactive content. Travel destinations can determine users’ interaction motivations based on the content and frequency of user posts on a platform, and can design interactive content around user engagement motivations to enhance social interaction.

Finally, FOC was found to moderate the relationship between SAT and TI. In other words, low FOC implies increased travelers’ satisfaction and travel intention. Therefore, to reduce tourists’ FOC, travel destinations must establish a staff health monitoring system and a daily registration of staff health status. Scenic public health and prevention and control measures should be implemented and cleanliness and hygiene in the scenic area should be maintained. Before important holidays and large events, inspections should be organized to promptly identify prevention and control loopholes and risk points, and rectifications and improvements should be ensured. Tourist places can implement time-sharing reservations using online booking and big data platforms and issue early warning alerts to hot scenic spots during peak tourist periods to reasonably regulate the flow of people. Younger groups are not significantly affected by FOC and can increase publicity to similar groups through channels such as social media and big data platforms.

### 5.4. Limitations and future research

Although the present study yields many interesting results, it has several limitations. First, all respondents in the survey are Chinese and the object of study is relatively homogeneous. Future studies could compare and analyze findings from other countries. Second, this study is a cross-sectional investigation. The analysis is done in a relatively short period of time. Future research could use a longitudinal approach to confirm causality and assess the consequences of the study’s framework over a long period of time. Third, this study only investigated the mediating effect in the relationship between PQ and TI, without examining the mediating effect in the relationship between PR, SI, and TI. Future studies could expand the research from this perspective. Forth, age is used as the control variable. However, there are significant differences in the characteristics of MZ and non-MZ eras in China. Therefore, future studies should divide age into MZ and non-MZ to draw clear insights on the moderating effect of age. Finally, in this study, fear of COVID-19 is analyzed as a moderating variable. Future research could consider fear of COVID-19 as an independent variable.

## 6. Conclusion

This study analyzes the relationship between perceived value antecedent variables, perceived quality, perceived risk, social interaction, outcome variable satisfaction, and travel intention in the context of the COVID-19 pandemic. It also verifies the mediating role of perceived value dimension and satisfaction. This study verifies the role of fear of COVID-19 as a moderating variable in the relationship between satisfaction and travel intention.

Although social interaction is a very important variable in the COVID-19 context, research on the topic is limited; hence, future studies could explore the impact of social interaction. In addition, existing literature is expanded by dividing perceived value into multiple dimensions.

The travel industry has experienced a decline in the number of travelers during the pandemic for various reasons, and the decline has negatively affected the industry’s profitability. However, as discussed by previous studies on COVID-19, fear of COVID-19 not only adds to the existing literature by recalibrating it to suit the Chinese situation but also by expanding it to the tourism field. The results of this study help the tourism industry overcome the difficulties caused by the pandemic and establish effective strategies to enable continuous growth.

## Data availability statement

The raw data supporting the conclusions of this article will be made available by the authors, without undue reservation.

## Ethics statement

The studies involving human participants were reviewed and approved by Changwon National University Institutional Review Board IRB Number: 7001066-202210-HR-066. Written informed consent for participation was not required for this study in accordance with the national legislation and the institutional requirements.

## Author contributions

All authors made a significant contribution to the work reported, whether that is in the conception, study design, execution, acquisition of data, analysis, and interpretation, or in all these areas; took part in drafting, revising, or critically reviewing the article; gave final approval of the version to be published; have agreed on the journal to which the article has been submitted; and agree to be accountable for all aspects of the work.

## Conflict of interest

The authors declare that the research was conducted in the absence of any commercial or financial relationships that could be construed as a potential conflict of interest.

## Publisher’s note

All claims expressed in this article are solely those of the authors and do not necessarily represent those of their affiliated organizations, or those of the publisher, the editors and the reviewers. Any product that may be evaluated in this article, or claim that may be made by its manufacturer, is not guaranteed or endorsed by the publisher.

## Supplementary material

The Supplementary material for this article can be found online at: https://www.frontiersin.org/articles/10.3389/fpsyg.2023.1136465/full#supplementary-material
