# Supplementary material for: Travel intentions of travelers in the COVID-19 context: The moderation of fear of COVID-19
Source: Front Psychol. 2023 Mar 2;14:1136465. doi: 10.3389/fpsyg.2023.1136465 (PMC10017471; doi:10.3389/fpsyg.2023.1136465)
Supplement: Supplementary file 1 [file Data_Sheet_1.pdf]

Appendix

Dimensions	Items	Source
Perceived emotional value （PVEM)	PVEM1 This travel destination is my favorite*	Peng et al. (2019) & Meeprom and Silanoi (2020)
	PVEM2 This travel destination makes me feel relaxed	
	PVEM3 This travel destination will make me feel good	
	PVEM4 This travel destination brings me happiness	
Perceived economic value （PVEC)	PVEC1 A reasonable price to pay for a tourist destination*	
	PVEC2 The price of additional expenses based on individual travel needs is acceptable	
	PVEC3 This travel destination is cost effective	
	PVEC4 Accurate pricing for this travel destination	
Perceived social value （PVSO)	PVSO1 Travel can make me more popular	
	PVSO2 Travel improves the way I see the world*	
	PVSO3 Traveling will leave a good impression on others	
	PVSO4 Helped to give me social recognition	
Perceived quality （PQ）	PQ1 This destination can offer a consistent quality tourism product	Chi et al. (2019)
	PQ2 This destination offers a quality experience	
	PQ3 I expect this destination will give me a good travel experience*	
	PQ4 The quality of this destination is better than other similar destinations	

Perceived risk （PR）	PR1 Disclosure of my personal information to travel companies, travel applications and web pages may be risky	Wang et al. (2019) & Şen Küpeli and Özer (2020)
	PR2 The quality of a tourist destination may not live up to its hype*	
	PR3 Travel puts me at potential risk of physical harm	
	PR4 Victims of travel accidents may not receive proper compensation*	
	PR5 All things considered, I would be concerned about suffering some kind of loss during the tour	
Social interaction (SI)	SI1 I maintain a close social relationship with my fellow travelers*	Lee et al. (2019) & Jasrotia et al. (2022)
	SI2 I spent a lot of time interacting with my fellow travelers	
	SI3 I often communicate with my fellow travelers	
	SI4 I will travel more if I can improve the relationship with fellow travelers during the trip	
Satisfaction (SAT)	SAT1 I am happy with my decision to visit this destination	Konuk (2019), Yan et al. (2021), & Liu et al. (2021)
	SAT2 I really like this travel destination	
	SAT3 I have positive feelings about this tourist destination*	
	SAT4 I made a wise choice in choosing this destination	
Travel intention （TI）	TI1 I may visit this destination again in the future	Chi et al (2019)
	TI2 I plan to visit this destination again in the future	
	TI3 I hope to visit this destination again	
Fear of	FOC1 I am afraid of contracting COVID while	Ahorsu et al.

COVID-19（FOC）	traveling-19	(2020)
	FOC2 The thought of COVID-19 when traveling makes me feel uncomfortable	
	FOC3 I am afraid of losing my life while traveling because of COVID-19	
	FOC4 While traveling, I get nervous or anxious when I see COVID-19 related news and stories on social media*	

*Questions are deleted during the formal test because of low reliability and appropriateness.
